# Increased Risk of Infections and Mortality Following Fractures in Multiple Myeloma: A Nationwide Population‐Based Retrospective Cohort Study

**DOI:** 10.1002/hsr2.72989

**Published:** 2026-08-04

**Authors:** Ching‐Chi Lee, Wei‐Yi Huang, Yu‐Fen Chen, I‐Szu Cheng, Kuo‐Yuan Huang

**Affiliations:** ^1^ College of Medicine, Center of Clinical Medical Research, National Cheng Kung University Hospital National Cheng Kung University Tainan Taiwan; ^2^ Department of Internal Medicine, National Cheng Kung University Hospital, College of Medicine National Cheng Kung University Tainan Taiwan; ^3^ Institute of Health and Welfare Policy National Yang Ming Chiao Tung University Taipei Taiwan; ^4^ Department of Healthcare Management Yuanpei University of Medical Technology Hsinchu Taiwan; ^5^ Department of Family Medicine, National Cheng Kung University Hospital, College of Medicine National Cheng Kung University Tainan Taiwan; ^6^ Department of Orthopedics, National Cheng Kung University Hospital, College of Medicine National Cheng Kung University Tainan Taiwan

**Keywords:** fractures, infections, mortality, multiple myeloma, National Health Insurance Research Database (NHIRD)

## Abstract

**Background and Aims:**

To determine whether patients with MM face higher risks of hospitalized infections and long‐term all‐cause mortality after a first fracture than non‐MM fracture patients at the population level.

**Methods:**

We performed a nationwide retrospective cohort study using Taiwan National Health Insurance Research Database from 2001 to 2019. Adults with MM and non‐MM adults with an index hospitalized fracture were identified and matched 1:10 by propensity scores. Cox proportional‐hazards models and Kaplan–Meier analyses compared risks of lower respiratory tract, reproductive or urinary tract, intra‐abdominal, and skin and soft‐tissue infections requiring hospitalization, as well as all‐cause mortality.

**Results:**

During 2001–2019, the incidence and age‐standardized incidence of MM in Taiwan were 6.0 and 5.4 per 100,000 people, respectively, and both increased over time. After propensity‐score matching, 303 MM patients with a first hospitalized fracture were compared with 3,030 non‐MM fracture patients. Compared with matched non‐MM patients, MM patients had significantly higher risks of lower respiratory tract infections (adjusted hazard ratio [AHR] = 2.12, 95% confidence interval [CI]: 1.72–2.61; *p* < 0.001), reproductive or urinary tract infections (AHR = 1.40, 95% CI: 1.06–1.86; *p* = 0.019), and all‐cause mortality (AHR = 1.98, 95% CI: 1.72–2.28; *p* < 0.001). The corresponding incidence rates per 1,000 person‐years were also higher in MM than non‐MM patients for lower respiratory tract infections (237.96 vs. 60.92), reproductive or urinary tract infections (107.35 vs. 41.26), skin and soft‐tissue infections (28.77 vs. 15.30), and all‐cause mortality (271.67 vs. 84.39). These associations were generally consistent across subgroups stratified by age, sex, and fracture type.

**Conclusion:**

MM was associated with substantially higher risks of serious infections and death after fracture. These findings support early multidisciplinary post‐fracture care, including infection surveillance and prevention, in MM patients.

## Summary

- MM patients with fractures face nearly double the risk of mortality compared to non‐MM patients.
- Hospitalization for respiratory and urinary infections is significantly higher in MM patients post‐fracture.
- A nationwide Taiwanese database and propensity score matching ensured robust cohort comparison.
- MM patients showed elevated infection risks across nearly all age, sex, and fracture type subgroups.
- Findings highlight the urgent need for multidisciplinary care in MM patients after fractures.

## 1 Introduction

Multiple myeloma (MM) is a malignant neoplasm of plasma cells in the bone marrow [1], accounts for approximately 1% of all cancers and 10% of hematologic malignancies [2]. For the overall population, the incidence of MM varies widely worldwide, from 0.54 to 5.3 per 100,000 people [3, 4]. MM is frequently associated with substantial bone destruction, such as osteolytic lesions, osteopenia/osteoporosis, and fractures [5, 6]. A population‐based US study found that MM patients have a ninefold increased risk of fractures compared to the general population [6], and these bone complications can be painful and substantially reduce the quality of life among MM individuals [7]. To mitigate skeletal‐related complications, bisphosphonates are recommended for most MM patients [1], and zoledronic acid treatment has been shown to improve MM prognosis [8]. However, in Taiwan, data on the epidemiological burden of MM, particularly its population‐based incidence and fracture risk, remain limited.

Patients with MM are at increased risk of fracture because malignant plasma cells disrupt normal bone remodeling by increasing osteoclast‐mediated bone resorption and suppressing osteoblast‐mediated bone formation, leading to osteolytic lesions, generalized bone loss, vertebral compression fractures, and pathologic fractures [5, 9]. In addition to substantial skeletal morbidity [5, 6], previous studies have shown that MM patients are at increased risks of infections, because fracture‐related tissue injury, immobilization, hospitalization, and possible orthopedic procedures provide opportunities for bacterial entry and nosocomial exposure [10, 11]. Notably, these risks are further amplified by myeloma‐related immunoparesis, impaired humoral immunity, bone marrow dysfunction, renal impairment, and treatment‐related immunosuppression [11, 12]. In two Swedish population‐based studies, MM was associated with a 5.3‐ to 7.1‐fold increased risk of developing infections compared with matched controls [13, 14]. Importantly, these infections may lead to mortality through progression to sepsis, septic shock, circulatory collapse, multiorgan dysfunction, and infection‐triggered cardiovascular compromise in this immunocompromised population [13, 14].

However, population‐level evidence specifically evaluating whether MM amplifies the risks of hospitalized infections and long‐term mortality following fracture episodes remain limited. Given the combined burden of MM‐related immune dysfunction, treatment‐related immunosuppression, skeletal fragility, and fracture‐related immobilization or hospitalization, we hypothesized that MM patients who sustain fractures would have higher subsequent risks of hospitalized infections and all‐cause mortality than fracture patients without MM. To test this hypothesis, we used a Taiwan nationwide database to compare long‐term infections and survival outcomes between MM and non‐MM patients after a first hospitalized fracture.

## 2 Materials and Methods

### 2.1 Data Source

Taiwan's National Health Insurance (NHI) program is compulsory, covering all citizens except incarcerated individuals. We identified eligible patients from the NHI Research Database (NHIRD), which includes data for 99% of inpatient and outpatient claims, encompassing demographics, diagnoses, procedures, pharmacy records, and prescription information for the over 23 million people [15]. The NHIRD thus provides a valuable resource for epidemiological and observational research. We utilized the longitudinal NHIRD dataset for 2,000,000 people enrolled before December 2000, representing approximately 9.35% of the Taiwanese population [15].

Our study was approved by the Institutional Review Board of our hospital (B‐EX‐112‐020), which waived the requirement for written informed consent due to the study's retrospective nature. The study was reported in accordance with the Strengthening the Reporting of Observational Studies in Epidemiology (STROBE) statement [16].

### 2.2 Patient Selection and Definition

In the retrospective cohort study, eligible participants were adults aged 18 years or older who experienced their first hospitalized fracture during 2001–2019. We excluded patients with missing data or incarcerated individuals who are not covered by the National Health Insurance program. These criteria yield two patient groups: (1) the MM group, consisting of MM patients with their first hospitalized fracture, and (2) the non‐MM group, comprising individuals without MM who experienced a hospitalized fracture during the study period.

Patients diagnosed with MM were identified using the International Classification of Diseases (ICD) codes 203.00, 203.01, or 203.02 (ICD‐9‐CM), or C90.00, C90.01, or C90.02 (ICD‐10‐CM), from outpatient or inpatient claims, or through the hemato‐oncological registration code (97323). The index date was defined as the date of this first fracture episode. The selection process is shown in Supplemental Figure 1.

**Figure 1:**
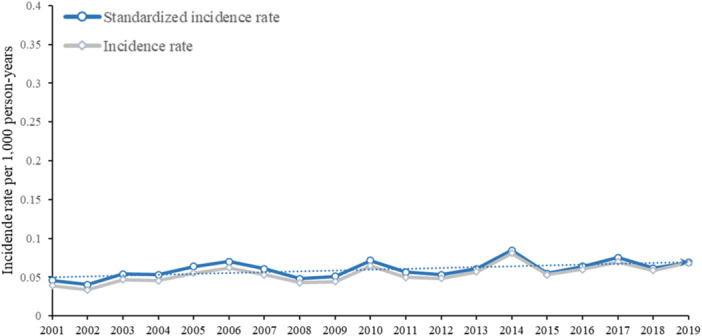
Incidence and age‐standardized incidence of multiple myeloma in Taiwan from 2001 to 2019. Incidence rates are presented as per 1,000 person‐years for visualization clarity.

### 2.3 Covariate Assessment

We characterized each patient's demographics and clinical attributes within 12 months before the index date, evaluating an array of prespecified covariates (e.g., demographic information, comorbidities, and Charlson comorbidity index), potentially associated with the outcome. We also evaluated hospitalized fracture type, as well as reduction and immobilization procedures during the index hospitalization. See Supplemental Tables 1 and 2 for the relevant ICD‐9‐CM and ICD‐10‐CM codes.

### 2.4 Outcome and Follow‐up

Both MM and non‐MM patients were followed from the index date until the first occurrence of any study outcome. Outcomes included all‐cause mortality and first hospitalization due to the following common infections: lower respiratory tract infections, intraabdominal infections, reproductive or urinary tract infections, and skin and soft‐tissue infections. We identified each outcome based on inpatient diagnosis codes listed in any position, or Taiwan Health Insurance Service claims codes (Supplementary Table 3). We confirmed death by linking each patient's records to the National Death Registry using unique identification numbers.

### 2.5 Statistical and Subgroup Analyses

Statistical analyses were performed using SAS version 9.3 (SAS Institute, Cary, NC, USA). All tests were 2‐sided, and *p* < 0.05 was considered statistically significant. Baseline characteristics between the MM and non‐MM patients were compared using standardized differences rather than significance testing, because standardized differences are less sensitive to sample size and are recommended for assessing covariate balance in the original (i.e., before matching) and matched cohorts; a value < 0.1 was considered to indicate adequate balance. The incidences of infections and all‐cause mortality per 1,000 person‐years were compared between MM and non‐MM patients using Poisson regression. A linear‐by‐linear association between calendar year and MM incidence was tested using Pearson's correlation.

Propensity scores (PSs) were estimated using multivariable logistic regression including prespecified covariates potentially associated with infections or mortality after fracture: age, ischemic heart disease, overweight or obesity, alcoholism‐associated disorders, neuropathy, hemato‐oncological disease other than MM, lymphoma or leukemia, anemia, hypercalcemia, end‐stage renal disease, chronic kidney disease, Charlson comorbidity index (CCI), fracture type, and fracture year. These variables were selected based on their clinical relevance and potential influence on infection or mortality risk in MM patients with fractures. Nearest‐neighbor matching without replacement was performed at a 1:10 ratio with a caliper width of 0.025 on the propensity‐score scale, as previously recommended [17].

Time‐to‐event outcomes, including hospitalized infections and all‐cause mortality, were visualized using Kaplan–Meier survival curves and analyzed using Cox proportional hazards models. Results are presented as hazard ratios (HRs) with 95% confidence intervals (CIs) in the original cohort (i.e., before matching) and as adjusted hazard ratios (AHRs) with 95% CIs in the matched cohort.

Prespecified subgroup analyses were conducted according to sex, age (< 65 vs ≥ 65 years), and fracture type (pathologic vs non‐pathologic; vertebral vs non‐vertebral). These analyses were considered exploratory in terms of effect heterogeneity and were interpreted cautiously.

## 3 Results

### 3.1 Study Population

During the 2001–2019 period, the incidence and age‐standardized incidence of MM in Taiwan were 6.0 and 5.4 per 100,000 people, respectively (Figure 1). Incidence rates were standardized to per 100,000 population, while Figure 1 is presented as per 1,000 person‐years for better visualization. Both measures showed a positive correlation with calendar year (r = 0.560, *p* = 0.01, and r = 0.681, *p* = 0.001), indicating an increasing trend over time.

Based on our inclusion and exclusion criteria, we identified 331 MM patients and 184,730 non‐MM patients in the longitudinal NHIRD dataset (Supplemental Figure 1). In the original cohort (i.e., before PS matching), MM patients were generally older, had higher insurance coverage, and higher CCI scores compared with non‐MM patients. They were also more likely to have anemia, ischemic heart disease, other hemato‐oncological diseases, lymphoma/leukemia, diabetes mellitus, chronic obstructive pulmonary disease, chronic kidney disease, end‐stage renal disease, cerebrovascular disease, neuropathy, congestive heart failure, alcoholism‐related disorders, systemic autoimmune disease, and hypercalcemia (Table 1, left). Furthermore, they were more likely to have pathologic or vertebral fractures but less likely to undergo surgical reduction or immobilization.

**Table 1:** Baseline demographics, comorbidities, and comorbid severity (measured within 365 days before the hospitalized fracture), and type and surgery of the hospitalized fracture among patients with and without multiple myeloma (MM) before and after propensity‐score matching.

	Original cohort			Matched cohort		
	MM (*n* = 331)	Non‐MM (*n* = 184,730)	Standardized difference	MM (*n* = 303)	Non‐MM (*n* = 3,030)	Standardized difference
Patient demographic						
Age, years			0.76			0.06
< 40	7 (2.11)	47,934 (25.95)		7 (2.31)	54 (1.78)	
40–65	131 (39.58)	68,809 (37.25)		118 (38.94)	1,097 (36.20)	
≥ 65	193 (58.31)	67,987 (36.80)		178 (58.75)	1,879 (62.01)	
Gender			0.03			0.01
Male	164 (49.55)	94,636 (51.23)		145 (47.85)	1,430 (47.19)	
Female	167 (50.45)	90,094 (48.77)		158 (52.15)	1,600 (52.81)	
Insurance (new Taiwan dollars per month)			0.15			0.13
< 20,000	121 (36.56)	79,578 (43.08)		114 (37.62)	1,265 (41.75)	
20,000–34,999	135 (40.79)	73,125 (39.58)		123 (40.59)	1,263 (41.68)	
≥ 35,000	75 (22.66)	32,027 (17.34)		66 (21.78)	502 (16.57)	
Smoking	3 (0.91)	2,818 (1.53)	0.06	3 (0.99)	61 (2.01)	0.08
Comorbidity						
Anemia	153 (46.22)	26,522 (14.36)	0.74	134 (44.22)	1,384 (45.68)	0.03
Ischemic heart disease	120 (36.25)	37,933 (20.53)	0.35	110 (36.30)	1,151 (37.99)	0.03
Chronic liver disease	106 (32.02)	38,591 (20.89)	0.25	96 (31.68)	981 (32.38)	0.01
Hemato‐oncology (excluding multiple myeloma)	97 (29.31)	15,311 (8.29)	0.56	87 (28.71)	961 (31.72)	0.07
Lymphoma or leukemia	20 (6.04)	533 (0.29)	0.33	18 (5.94)	219 (7.23)	0.05
Diabetes mellitus	95 (28.70)	40,577 (21.97)	0.16	87 (28.71)	1,060 (34.98)	0.13
Chronic obstructive pulmonary disease	85 (25.68)	30,491 (16.51)	0.23	78 (25.74)	893 (29.47)	0.08
Chronic kidney disease	68 (20.54)	10,978 (5.94)	0.44	58 (19.14)	497 (16.40)	0.07
End‐stage renal disease	19 (5.74)	2,631 (1.42)	0.23	17 (5.61)	117 (3.86)	0.08
Cerebrovascular disease	66 (19.94)	29,348 (15.89)	0.11	60 (19.80)	867 (28.61)	0.21
Neuropathy	64 (19.34)	21,314 (11.54)	0.22	57 (18.81)	583 (19.24)	0.01
Congestive heart failure	63 (19.03)	16,694 (9.04)	0.29	58 (19.14)	514 (16.96)	0.06
Alcoholism‐associated disorders	48 (14.50)	16,144 (8.74)	0.18	42 (13.86)	449 (14.82)	0.03
Systemic autoimmune diseases	34 (10.27)	9,845 (5.33)	0.19	30 (9.90)	288 (9.50)	0.01
Hypercalcemia	32 (9.67)	150 (0.08)	0.46	6 (1.98)	29 (0.96)	0.009
Overweight or obesity	2 (0.60)	1,910 (1.03)	0.05	2 (0.66)	9 (0.30)	0.05
Charlson comorbidity index			0.80			0.02
0	29 (8.76)	68,753 (37.22)		28 (9.24)	282 (9.31)	
1–2	94 (28.40)	57,863 (31.32)		85 (28.05)	815 (26.90)	
≥ 3	208 (62.84)	58,114 (31.46)		190 (62.71)	1,933 (63.80)	
Hospitalized facture						
Type						
Pathologic fracture	271 (81.87)	16,500 (8.93)	2.15	248 (81.85)	514 (16.96)	1.71
Vertebral fracture	61 (18.43)	21,723 (11.76)	0.19	59 (19.47)	403 (13.30)	0.17
Surgery						
Reduction	42 (12.69)	82,131 (44.46)	0.75	38 (12.54)	1,142 (37.69)	0.61
Immobilization	0 (0.00)	1304 (0.71)	0.12	0 (0.00)	21 (0.69)	0.12

After matching, the cohort included 303 MM patients and 3,030 non‐MM patients. The age range was 19–82 years in the MM group and 18–86 years in the non‐MM group. All standardized differences for covariates fell below 0.1, indicating that the two groups were well balanced (Table 1, right).

### 3.2 Infection Incidence and Mortality Rate Between MM and Non‐MM Patients

Table 2 shows the follow‐up duration, number of events, and incidence rates before and after PS matching. Median follow‐up time varied, ranging from 0.83 to 6.89 years, depending on treatment group and outcome. In the matched cohort, patients with MM had higher absolute incidences of lower respiratory tract infections (237.96 vs 60.92 per 1,000 person‐years; *p* < 0.001), reproductive or urinary tract infections (107.35 vs 41.26 per 1,000 person‐years; *p* < 0.001), and skin and soft‐tissue infections (28.77 vs 15.30 per 1,000 person‐years; *p* < 0.001), as well as a higher all‐cause mortality rate (271.67 vs 84.39 per 1,000 person‐years; *p* < 0.001), than non‐MM patients. In Cox proportional‐hazards models, MM was associated with higher risks of lower respiratory tract infections (AHR = 2.12, 95% CI: 1.72–2.61; *p* < 0.001), reproductive or urinary tract infections (AHR = 1.40, 95% CI: 1.06–1.86; *p* = 0.019), and all‐cause mortality (AHR = 1.98, 95% CI: 1.72–2.28; *p* < 0.001).

**Table 2:** Follow‐up duration, number of cases, and incidences of outcomes before and after propensity score matching.

	Original cohort		Matched cohort	
	MM (*n* = 331)	Non‐MM (*n* = 184,730)	MM (*n* = 303)	Non‐MM (*n* = 3,030)
Lower respiratory tract infections				
Total follow‐up person‐year	633	1334737	605	14296
Median follow‐up year [IQR]	0.83 [0.27, 2.49]	6.20 [2.33, 11.56]	0.86 [0.27, 2.61]	3.29 [0.87, 7.21]
Number of cases	164	34353	144	871
Incidence rate per 1,000 person‐year (95% CI)	258.97 (220.85, 301.78)	25.74 (25.47, 26.01)	237.96 (200.68, 280.15)	60.92 (56.95, 65.11)
Intraabdominal infections				
Total follow‐up person‐year	813	1389601	764	15369
Median follow‐up year [IQR]	1.43 [0.46, 3.60]	6.62 [2.72, 11.85]	1.49 [0.48, 3.66]	3.87 [1.23, 7.75]
Number of cases	15	8283	11	167
Incidence rate per 1,000 person‐year (95% CI)	18.45 (10.33, 30.43)	5.96 (5.83, 6.09)	14.41 (7.19, 25.78)	10.87 (9.28, 12.65)
Reproductive or urinary tract infections				
Total follow‐up person‐year	705	1309696	671	14032
Median follow‐up year [IQR]	0.99 [0.28, 2.80]	5.99 [2.17, 11.42]	1.04 [0.30, 2.84]	3.15 [0.85, 7.06]
Number of cases	78	25814	72	579
Incidence rate per 1,000 person‐year (95% CI)	110.69 (87.49, 138.14)	19.71 (19.47, 19.95)	107.35 (84.00, 135.19)	41.26 (37.97, 44.76)
Skin and soft tissue infections				
Total follow‐up person‐year	784	1354405	730	14964
Median follow‐up year [IQR]	1.30 [0.45, 3.44]	6.38 [2.48, 11.65]	1.38 [0.46, 3.44]	3.64 [1.10, 7.52]
Number of cases	21	13238	21	229
Incidence rate per 1,000 person‐year (95% CI)	26.79 (16.58, 40.94)	9.77 (9.61, 9.94)	28.77 (17.81, 43.98)	15.30 (13.39, 17.42)
Overall mortality				
Total follow‐up person‐year	838	1423331	784	15855
Median follow‐up year [IQR]	1.48 [0.48, 3.69]	6.89 [2.89, 12.11]	1.51 [0.51, 3.71]	4.07 [1.33, 8.05]
Number of cases	233	47973	213	1338
Mortality rate per 1,000 person‐year (95% CI)	278.01 (243.46, 316.09)	33.70 (33.40, 34.01)	271.67 (236.41, 310.71)	84.39 (79.93, 89.03)

Abbreviations: CI, confidence interval; IQR, interquartile range; MM, multiple myeloma.

### 3.3 Association between Infections or Mortality and MM Patients

**Figure 2:**
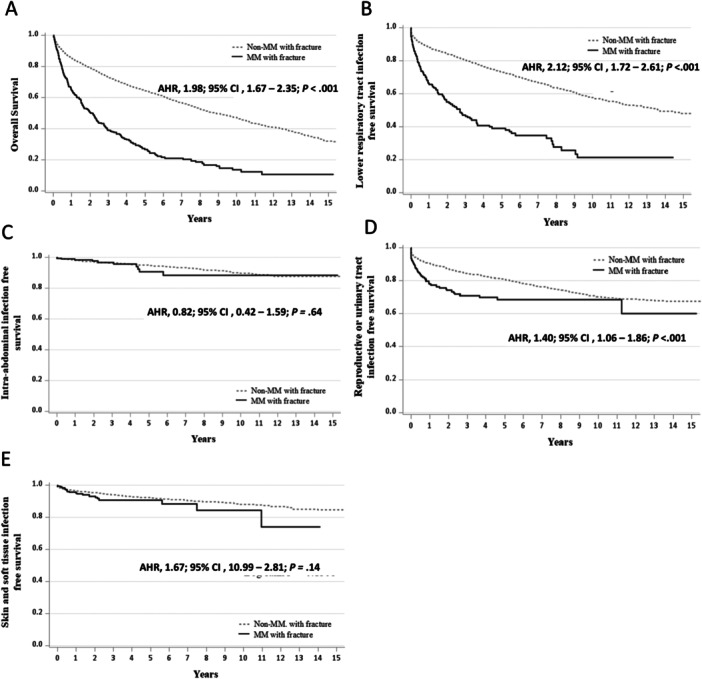
Kaplan–Meier curves comparing patients with and without multiple myeloma (MM) after the index fracture in the matched cohort for: (A) overall mortality, (B) hospitalization due to lower respiratory tract infections, (C) hospitalization due to intra‐abdominal infections, (D) hospitalization due to reproductive or urinary tract infections, (E) hospitalization due to skin and soft‐tissue infections. Adjusted hazard ratios (AHRs) were estimated using Cox proportional‐hazards models.

In the matched cohort, MM patients were associated with a higher hazard of all‐cause mortality (AHR, 1.98; 95% CI, 1.72–2.28; Figure 2A), lower respiratory tract infections (AHR, 2.12; 95% CI, 1.72–2.61; Figure 2B), and reproductive or urinary tract infections (AHR, 1.40; 95% CI, 1.06–1.86; Figure 2D), compared with non‐MM patients.

### 3.4 Subgroup Analysis

**Figure 3:**
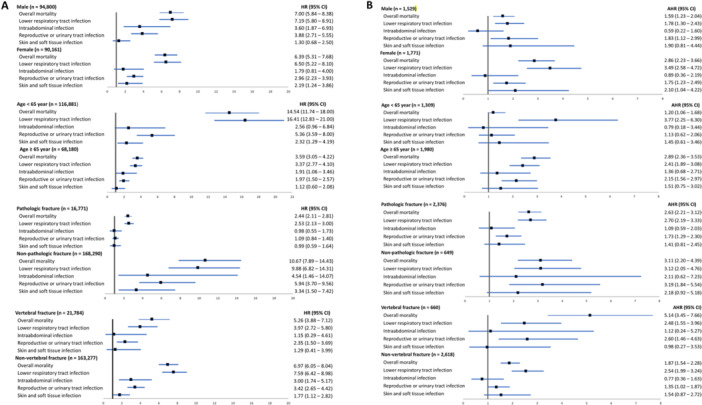
Hazard ratios (HRs, Figure 3A) and adjusted hazard ratios (AHRs, Figure 3B) and 95% confidence intervals for assessing the association between outcomes after the index fracture and MM patients among subgroup patients in the original and matched cohort, respectively, compared with non‐MM patients. HRs and AHRs were estimated using Cox proportional‐hazards models.

In the original cohort, HRs of different infections and overall mortality were evaluated across subgroups defined by sex, age, and fracture types (Figure 3A). Compared with non‐MM patients, MM patients showed significantly increased risks of overall mortality and lower respiratory tract infections across all subgroups. MM patients also had a significantly higher risk of reproductive or urinary tract infections in all subgroups except those with pathologic fractures.

In the matched cohort, AHRs for infections and overall mortality by subgroup were analyzed (Figure 3B). Relative to non‐MM patients, MM patients had significantly higher risks of lower respiratory tract infections in all subgroups. Additionally, they had significantly higher overall mortality across all subgroups, except for those younger than 65, and higher reproductive or urinary tract infection risks in all subgroups, except for those younger than 65 and those with non‐vertebral fractures.

## 4 Discussion

The incidence of MM varies widely worldwide, from 0.54 to 5.3 per 100,000 people [3, 4]. In line with these global figures and prior studies showing a consistent rise in MM incidence [3] our data revealed a year‐related upward trend, with a standardized incidence of 5.1 per 100,000 in Taiwan. Moreover, MM patients following the first hospitalized fracture had significantly higher risks of overall mortality and hospitalization for lower respiratory tract infections than non‐MM patients in our overall cohort, and these associations were consistent across most subgroup analyses. Moreover, MM patients had a significantly increased risk of reproductive or urinary tract infections, although this association was attenuated in patients under 65 years.

Patients with MM are particularly vulnerable to infections and early mortality through several overlapping mechanisms. First, disease‐related immune dysfunction includes suppression of normal polyclonal immunoglobulin production, impaired humoral immunity, reduced complement activity, and impaired host defense against encapsulated bacteria [1, 18]. As a result, MM patients are predisposed to infections by encapsulated bacteria (e.g., *Streptococcus pneumoniae* [19] and *Haemophilus influenzae* [20]), common causes of lower respiratory tract infections. Second, anti‐myeloma treatments, corticosteroids, stem cell transplantation, repeated relapse, and salvage therapies may produce cumulative immunosuppression [18]. Third, MM patients also frequently have anemia, renal dysfunction, hypercalcemia, cardiovascular disease, frailty, and reduced functional reserve, which may further increase the risk of clinical deterioration after acute events [21, 22]. Finally, following fracture, immobilization, hospitalization, surgical or supportive procedures, impaired pulmonary toileting, pain‐related hypoventilation, urinary retention, and catheter exposure may further predispose these patients to lower respiratory tract, urinary tract [23, 24], skin and soft‐tissue, and other serious infections [25, 26]. Once serious infection develops, progression to sepsis, septic shock, acute respiratory failure, acute kidney injury, hemodynamic instability, cardiovascular decompensation, and multiorgan failure may occur [13, 14]. These pathways provide a plausible biological explanation for the increased infection‐related hospitalization and all‐cause mortality observed in MM patients after fracture.

Several factors may explain the temporal trends observed in this nationwide cohort. First, the incidence and age‐standardized incidence of MM in Taiwan increased from 2001 to 2019, consistent with global observations of a rising MM burden [3, 4]. This increase might partly reflect population aging, improved diagnostic awareness, broader use of laboratory and imaging evaluations, and changes in MM diagnostic criteria that facilitate earlier or more complete case detection [1, 2]. Second, advances in anti‐myeloma therapy, including proteasome inhibitors, immunomodulatory drugs, monoclonal antibodies, bisphosphonates, and stem cell transplantation, have improved disease control and survival [1, 8]. As a result, more patients may live long enough to develop skeletal‐related events, including vertebral compression fractures, pathologic fractures, and other fracture‐related complications [5, 6, 9]. Third, although new therapies have improved MM outcomes, they may also contribute to cumulative immune dysfunction through treatment‐related immunosuppression, corticosteroid exposure, repeated relapse, and salvage therapies, thereby increasing susceptibility to bacterial, viral, and fungal infections [10, 11, 12, 18, 27]. Finally, improvements in imaging, fracture diagnosis, orthopedic management, and claims‐based coding practices over time may also have influenced the observed patterns of fracture and infection‐related hospitalization [15, 25, 26]. Therefore, the increasing MM burden over the study period suggests that fracture‐associated infections and post‐fracture mortality may become increasingly important clinical issues in Taiwan. Future studies should evaluate whether structured post‐fracture care pathways, early infection surveillance, vaccination strategies, antimicrobial prophylaxis in selected high‐risk patients, pulmonary and urinary complication prevention, rehabilitation, and coordinated hematology–orthopedic care can reduce infection‐related hospitalization and mortality.

We acknowledge a few limitations. First, previous research has demonstrated that cardiovascular disease is a key determinant of overall survival in MM [21, 22, 28]. Although we did not classify infection‐related mortality, it is reasonable to conclude that patients with MM who experience fractures have substantially poorer prognoses than their non‐MM counterparts, given that MM‐related immunodeficiency itself and newer MM therapies may further increase the risk of infections [27]. Second, although we adjusted for many potential confounders, residual confounding (e.g., body mass index, smoking duration) may remain. Third, despite our attempts to balance the two cohorts, MM patients could have more severe fractures and frailty overall. Fourth, the low incidence of certain infections and limited sample sizes in subgroups may reduce precision. Fifth, subgroup analyses generally showed consistent associations across clinically relevant strata; however, these analyses were exploratory and should be interpreted cautiously because these analyses are susceptible to multiplicity and potential false‐positive results. Finally, while we achieved baseline comparability using PS matching, we did not assess changes in quality of life or frailty during the follow‐up period. Despite these limitations, our interpretation of the findings emphasized the magnitude and precision of the estimated associations, as reflected by hazard ratios, 95% confidence intervals, and absolute incidence rates, rather than relying solely on statistical significance testing.

## 5 Conclusions

MM patients following their first hospitalization for fracture had significantly higher risks of all‐cause mortality and hospitalization for lower respiratory tract infections and reproductive or urinary tract infections than non‐MM patients with fracture. These findings support early multidisciplinary post‐fracture care, proactive infection prevention, and close clinical monitoring in this high‐risk population.

## Author Contributions

**Ching‐Chi Lee:** writing – original draft, formal analysis, investigation, data curation. **Wei‐Yi Huang:** formal analysis, visualization, methodology. **Yu‐Fen Chen:** formal analysis, visualization, methodology. **I‐Szu Cheng:** conceptualization, formal analysis. **Kuo‐Yuan Huang:** writing – review and editing, conceptualization, funding acquisition, supervision, data curation, formal analysis, investigation, validation.

## Disclosure

All authors have read and approved the final version of the manuscript Dr. Kuo‐Yuan Huang had full access to all of the data in this study and takes complete responsibility for the integrity of the data and the accuracy of the data analysis.

## Ethics Statement

The study was approved by the Institutional Review Board of National Cheng Kung University Hospital (B‐EX‐112‐020), and the requirement of obtaining informed consent was waived.

## Conflicts of Interest

The authors declare no conflicts of interest.

## Transparency Statement

Prof. Kuo‐Yuan Huang affirms that this manuscript is an honest, accurate, and transparent account of the study being reported; that no important aspects of the study have been omitted; and that any discrepancies from the study as planned have been explained.

## Supporting information


Supporting File

## Data Availability

The data that support the findings of this study are subject to third‐party restrictions and are not publicly available. The data were obtained from the Health and Welfare Data Science Center (HWDC), Ministry of Health and Welfare, Taiwan. Researchers who meet the criteria for access to confidential data may apply for access through the HWDC, subject to approval by the relevant authorities.
