# Supplementary material for: Increased Risk of Infections and Mortality Following Fractures in Multiple Myeloma: A Nationwide Population‐Based Retrospective Cohort Study
Source: Health Sci Rep. 2026 Aug 4;9(8):e72989. doi: 10.1002/hsr2.72989 (PMC13438894; doi:10.1002/hsr2.72989)
Supplement: Supplementary file 1 [file HSR2-9-e72989-s001.docx]

**Supplemental Figure and Table**

**Supplemental Figure 1**. Flow chart for patient selection.

**Supplemental Table 1.** International Classification of Diseases, 9th or 10th Revision, Clinical Modification (ICD-9-CM or ICD-10-CM) diagnosis codes for hospitalized fracture and surgery

| Variables | Diagnosis codes |
| --- | --- |
| **Categories of fracture** | |
| Pathologic | ICD-9-CM: 733.1  CD-10-CM: M80.0-M80.8, M84.3, M84.4, M84.5, M84.6, M48.5 |
| Vertebral | ICD-9-CM: 805, 806  ICD-10-CM: S12, S22.0, S32.0, |
| Scapula or shoulder | ICD-9-CM: 811  ICD-10-CM: S42.1, S42.9 |
| Hip | ICD-9-CM: 822  ICD-10-CM: S72.0 |
| Femur | ICD-9-CM: 820, 821  ICD-10-CM: S72, S791 |
| Humerus | ICD-9-CM: 812  ICD-10-CM: S42.2, S42.3, S42.4, S49.1 |
| Forearm | ICD-9-CM: 813  ICD-10-CM: S52.0-52.9, S59.0, S59.1, S59.2 |
| Hand and wrist | ICD-9-CM: 814, 815, 816, 817, 818  ICD-10-CM: S62.0-62.9 |
| Rib | ICD-9-CM: 807, 810  ICD-10-CM: S22.3, S42.0 |
| Pelvis | ICD-9-CM: 808  ICD-10-CM: S32.1-32.89 |
| Tibia or Fibula | ICD-9-CM: 823  ICD-10-CM: S82.1-S82.9, S89.0-S89.3 |
| Ankle | ICD-9-CM: 824  ICD-10-CM: S82.5 |
| Foot | ICD-9-CM: 825, 826  ICD-10-CM: S92.0-S92.9 |
| Extremities | ICD-9-CM: 819, 828, 814, 815, 816, 817, 818, |
| Others | ICD-9-CM: 809, 829 |
| **Surgery** | |
| Reduction | Order codes: 64028C, 64029B, 64030B, 64031C, 64032B, 64271C, 64034B, 64035C, 64036C,64041C,64042C, 64043C, 64044C, 64045C, 64046C, 64047C, 64048C, 64049C, 64160B: |
| Immobilization | Order codes: 64016C, 64271C, 64058B, 64178B, 64179B, 64180B, 64181B, 64182B, 64183B, 64281B |

**Supplemental Table 2.** International Classification of Diseases, 9th or 10th Revision, Clinical Modification (ICD-9-CM or ICD-10-CM) diagnosis or procedure codes for patient demographics or comorbidities

| Variables | Diagnosis or procedure codes |
| --- | --- |
| Smoking | ICD-9-CM diagnosis codes: 305.1, 649.0, 989.84, V15.82  ICD-10-CM diagnosis codes: F17.200, F17.201, F17.210, F17.211, F17.220, F17.221, F17.290, F17.291, T65.211A, T65.212A, T65.213A, T65.214A, T65.221A, T65.222A, T65.223A, T65.224A, T65.291A, T65.292A, T65.293A, T65.294A, Z87.891 |
| Systemic autoimmune diseases | ICD-9-CM diagnosis codes: 696.0, 696.1, 710.0, 710.1, 710.2, 710.3, 710.4, 714.0  ICD-10-CM diagnosis codes: L40, M05.7, M05.8, M05.9, M06.0, M06.1, M06.2, M06.3, M06.8, M06.9, M32, M33, M34, M35 |
| Neuropathy | ICD-9-CM diagnosis codes: 356.9, 250.6, 358.1, 951.0, 951.1, 951.3, 354.0-355.9, 713.5, 357.2  ICD-10-CM diagnosis codes: E08.4, E09.4, E10.4, E11.4, E13.4, G60.9, G73.3, H49.0, H49.1, H49.2 |
| Alcoholism-associated disorders | ICD-9-CM diagnosis codes: 696.0, 696.1, 710.0, 710.1, 710.2, 710.3, 710.4, 714.0, 283.0, 245.2, 340, 358.0, 364.0, 364.3, 555, 556  ICD-10-CM diagnosis codes: L40, M05.7, M05.8, M05.9, M06.0, M06.1, M06.2, M06.3, M06.8, M06.9, M32, M33, M34, M35, D59.0, D59.1, E06.3, G35, G70.0, H20.0, H20.9, K50, K51 |
| Overweight or obesity | ICD-9-CM diagnosis codes: 278.0, 278.00, 278.01, 278.02  ICD-10-CM diagnosis codes: E66 |
| Ischemic heart disease | ICD-9-CM diagnosis codes: 411, 413, 414  ICD-10-CM diagnosis codes: I20, I24, I25 |
| Cerebrovascular disease | ICD-9-CM diagnosis codes: 430-438  ICD-10-CM diagnosis codes: G45, G46, I60-I69 (Excluded I67.3, I67.83) |
| Congestive heart failure | ICD-9-CM diagnosis codes: 428, 398.91, 402.01, 402.11, 402.91, 404.01, 404.11, 404.91, 404.03, 404.13, 404.93  ICD-10-CM diagnosis codes: I09.81, I11.0, I13.0, I13.2, I50 |
| Chronic obstructive pulmonary disease | ICD-9-CM diagnosis codes: 491, 492, 496  ICD-10-CM diagnosis codes: J41, J42, J43, J44 |
| Chronic kidney disease | ICD-9-CM diagnosis codes: 403.00, 403.01, 403.10, 403.11,403.90, 403.91, 404.00, 404.01, 404.02,404.03,404.10,404.11, 404.12,404.13,404.90,404.91,404.92,404.93,585,V45.1,V56.0,V56.8  ICD-10-CM diagnosis codes: I12, I13, N18, Z49.3, Z99.2  ICD-9-CM procedure codes: 39.95, 54.98  ICD-10-CM procedure codes: 3E1M39Z, 5A1D00Z, 5A1D60Z |
| End-stage renal disease | Order code: 8001C, 58027C, 58029C, 58019C, 58020C,58021C, 58022C, 58023C, 58024C, 58025C, 58007C, 58007CA, 58007CB, 58014C, 58018C, 58030B, 58002CA, 58002CB, 58002C, 58009A, 58009B, 58010A, 58010B, 58011A, 58011AB, 58011B, 58011C, 58012A, 58012B, 58017B, 58017C, 58028C, 58026C |
| Chronic liver disease | ICD-9-CM diagnosis codes: 070.2, 070.3, 070.41, 070.44, 070.51, 070.54, V02.61, V02.62, 571.0, 571.1, 571.2, 571.3, 571.4, 571.5, 571.6, 571.8  ICD-10-CM diagnosis codes: B16, B17.0, B17.1, B18.0, B18.1, B18.2, B19.1, B19.2, K70, K73, K74, K75.4, K75.81, Z22.51, Z22.52 |
| Anemia | ICD9 diagnosis codes : 280, 280.0, 280.1, 280.8, 280.9, 281, 281.0, 281.1, 281.2, 281.3, 281.4, 281.8, 281.9, 282.0, 282.1, 282.2, 282.3, 282.4, 282.5, 282.60, 282.61, 282.62, 282.63, 282.69, 282.7, 282.8, 282.9, 283, 283.0, 283.10, 283.11, 283.19, 283.2, 283.9, 284, 284.0, 284.8, 284.9, 285, 285.0, 285.1, 285.2, 285.21, 285.22, 285.29, 285.8, 285.9  ICD10 diagnosis codes: D50, D50.0, D50.1, D50.8, D50.9, D51.0, D51.2, D51.3, D51.8, D51.9, D52.0, D52.1, D52.8, D52.9, D53.0, D53.1, D53.2, D53.8, D53.9, D55.0, D55.1, D55.2, D55.3, D55.8, D55.9, D56.0, D56.1, D56.2, D56.3, D56.5, D56.8, D56.9, D57.1, D57.00, D57.01, D57.02, D57.03, D57.09, D57.20, D57.211, D57.212, D57.213, D57.218, D57.219 D57.40, D57.42, D57.44, D57.411, D57.412, D57.413, D57.418, D57.419, D57.4312, D57.432, D57.433, 57.438, D57.439, D57.451, D57.452, D57.453, D57.458, D57.459, D58.0, D58.1, D57.811, D57.812, D57.813, D57.818, D57.819, D59.0, D59.1, D59.2, D59.3, D59.4, D59.5, D59.6, D59.8, D59.9, D60.0, D60.1, D60.8, D60.9, D61.01, D61.09, D61.1, D61.2, D61.3, D61.810, D61.811, D61.818, D61.81, D61.82, D61.89, D61.9, D62, D63, D63.0, D63.1, D63.8, D64.0, D64.1, D64.2, D64.3, D64.4, D64.8, D64.89, D64.9 |
| Hypercalcemia | ICD9 diagnosis codes: 27542  ICD10 diagnosis codes: E8352 |
| Hemato-oncology (excluding multiple myeloma) | ICD9 diagnosis codes: 140-208 (excluding 203.00, 203.01,203.02)  ICD10 diagnosis codes: C00-C97 (excluding C90.00, C90.01, C90.02) |
| Leukemia | ICD9 diagnosis codes: 204-208  ICD10 diagnosis codes: C91-C95 |
| Lymphoma | ICD9 diagnosis codes: 201-203 (excluding 203.00, 203.01,203.02)  ICD10 diagnosis codes: C81-C90, C96.9, C96.Z (excluding C90.00, C90.01, C90.02) |
| Diabetes mellitus | ICD9 diagnosis codes: 250  ICD10 diagnosis codes: E08, E09, E10, E11, E12, E13 |

**Supplemental Table 3**. International Classification of Diseases, 9^th^ or 10^th^ Revision, Clinical Modification (ICD-9-CM or ICD-10-CM) diagnosis codes^*^ used to identify study outcomes

| Outcomes | Diagnosis codes |
| --- | --- |
| Lower respiratory tract infection | ICD-9-CM: 480, 487, 481, 482, 483, 485, 486, 510, 513  ICD-10-CM: J09, J10, J11, J12, J13, J14, J15, J16, J17, J18, J85, J86 |
| Intraabdominal infection | ICD-9-CM: 572.0 (Excluding 006.3), 574.0, 574.1, 574.3, 574.4, 574.6, 574.7, 574.8, 575, 575.0, 575.1, 575.4, 576.1, 562.10, 562.11, 562.12, 562.13, 008.45  ICD-10-CM: K75.0 (Excluding A06.4), K80.0, K80.1, K80.3, K80.4, K80.6, K81, K82.2, K83.0, K57.0, K57.2, K57.32, K57.33, K57.4, K57.8, K57.92, K57.93 |
| Reproductive or urinary tract infection | ICD-9-CM: 590, 599.0, 601, 604, 614, 615, 616  ICD-10-CM: N10, N30, N41, N45, N70, N71, N72, N73, N74 |
| Skin and soft tissue infection | ICD-9-CM: 6806, 6807, 68110, 68111, 6826, 6827, 683, 68600, 68601, 6868, 6869, 70706, 70707, 70710,70711, 70712, 70713, 70714, 70715, 70719, 0400, 7854, 44024, 44023, 73005, 73006, 73007, 73015, 73016, 73017, 73025, 73026, 73027, 73035, 73036, 73037, 73075, 73076, 73077, 73085, 73086, 73087, 73095, 73096, 73097  ICD-10-CM: L97101, L97102, L97103, L97104, L97105, L97106, L97108, L97109, L97111, L97112, L97113, L97114, L97115, L97116, L97119, L97121, L97122, L97123, L97124, L97125, L97126, L97128, L97129, L97201, L97202, L97203, L97204, L97205, L97206, L97208, L97209, L97211, L97212, L97213, L97214, L97215, L97216, L97218, L97219, L97221, L97222, L97223, L97224, L97225, L97226, L97228, L97229, L97301, L97302, L97303, L97304, L97305, L97306, L97308, L97309, L97311, L97312, L97313, L97314, L97315, L97316, L97318, L97319, L97321, L97322, L97323, L97324, L97325, L97326, L97328, L97329, L97401, L97402, L97403, L97404, L97405, L97406, L97408, L97411, L97412, L97413, L97414, L97415, L97416, L97418, L97419, L97421, L97422, L97423, L97424, L97425, L97426, L97428, L97429, L97501, L97502, L97503, L97504, L97505, L97506, L97508, L97509, L97511, L97512, L97513, L97514, L97515, L97516, L97518, L97519, L97521, L97522, L97523, L97524, L97525, L97526, L97528, L97529, L97801, L97802, L97803, L97804, L97805, L97806, L97808, L97809, L97811, L97812, L97813, L97814, L97815, L97816, L97818, L97819, L97821, L97822, L97823, L97824, L97825, L97826, L97828, L97829, L97901, L97902, L97903, L97904, L97905, L97906, L97908, L97909, L97911, L97912, L97913, L97914, L97915, L97916, L97918, L97919, L97921, L97922, L97923, L97924, L97925, L97926, L97928, L97929, L89500, L89501, L89502, L89503, L89504, L89509, L89510, L89511, L89512, L89513, L89514, L89519, L89520, L89521, L89522, L89523, L89524, L89529, L89600, L89601, L89602, L89603, L89604, L89609, L89610, L89611, L89612, L89613, L89614, L89619, L89620, L89621, L89622, L89623, L89624, L89629, L02415, L02416, L02425, L02426, L02435, L02436, L02611, L02612, L02619, L02621, L02622, L02629, L02631, L02632, L02639, L03031, L03032, L03039, L03041, L03042, L03049, L03115, L03116, L03125, L03126, L043, L080, L081, L0881, L0882, L0889, L089, A480, I96, I70269, I7025, E1152, E11621, M86051, M86052, M86059, M86061, M86062, M86069, M86071, M86072, M86079, M86159, M86161, M86162, M86169, M86171, M86172, M86179, M86251, M86252, M86259, M86261, M86262, M86269, M86271, M86272, M86279, M86351, M86352, M86359, M86361, M86362, M86369, M86371, M86372, M86379, M86451, M86452, M86459, M86461, M86462, M86469, M86471, M86472, M86479, M86551, M86552, M86559, M86561, M86562, M86569, M86571, M86572, M86579, M86651, M86652, M86659, M86661, M86662, M86669, M86671, M86672, M86679, M868X5, M868X6, M868X7 |

^*^Based on any positions on inpatient claims.
